# Renal abscess complicating acute pyelonephritis in children: Two cases report and literature review

**DOI:** 10.1097/MD.0000000000036355

**Published:** 2023-12-01

**Authors:** Zhuqin Zhan, Xiaoliang Lin, Guangbo Li, Jinhua Zeng, Dequan Su, Jianying Liao, Qian Shen

**Affiliations:** a Department of Nephrology, Children’s Hospital of Fudan University Xiamen Branch (Xiamen Children’s Hospital), Xiamen, Fujian, China; b Department of Respiratory, Children’s Hospital of Fudan University Xiamen Branch (Xiamen Children’s Hospital), Xiamen, Fujian, China; c Department of Nephrology, Children’s Hospital of Fudan University, Shanghai, China.

**Keywords:** acute pyelonephritis, children, metagenomic next-generation sequencing, renal abscess

## Abstract

**Rationale:**

To describe the diagnostic and treatment approaches of renal abscesses complicated with acute pyelonephritis in children.

**Patient concerns:**

Two children presented with fever, vomiting, and abdominal pain with no typical manifestations, like frequent urination, urgency, dysuria, hematuria, foam urine, and lumbago. Renal abscess complicating acute pyelonephritis was diagnosed by B-ultrasound and computed tomography enhancement. Moreover, inflammatory markers were elevated significantly, but routine blood and urine cultures were repeatedly negative. The empirical anti-infection therapy had no obvious effect. A pathogenic diagnosis was confirmed in case two, and macro gene detection in blood and urine guided the follow-up treatment.

**Diagnoses:**

Both children were diagnosed with acute gastroenteritis on admission, but renal abscess complicating acute pyelonephritis were diagnosed by imaging examination.

**Interventions:**

Both children were given anti-infection therapy of third-generation cephalosporin, which had no obvious effect. Routine blood and urine cultures were repeatedly negative. Case one was changed to piperacillin sodium tazobactam. We further carried out blood and urinary metagenomic next-generation sequencing detection for case two. Meanwhile, meropenem and linezolid anti-infection treatment was given. The results showed overlapping infection with *Escherichia coli* and *Enterococcus faecalis*. According to the genetic test results, amoxicillin clavulanate potassium combined with nitrofurantoin were prescribed after discharge.

**Outcomes:**

Clinical symptoms of the 2 children disappeared, the infection was controlled, and imaging showed that renal abscess complicated with acute pyelonephritis disappeared.

**Lessons:**

The clinical spectrum of renal abscess complicating acute pyelonephritis is vague, with no specific manifestations, and can be easily misdiagnosed. B-ultrasound and computed tomography enhancement are helpful in making a definite diagnosis. Moreover, the sensitivity of routine culture is low, and metagenomic next-generation sequencing might be helpful to detect pathogenic microorganisms and guided treatment. Early treatment with broad-spectrum antibiotics might have favorable outcomes.

## 1. Introduction

Kidney abscess in children is a rare but serious condition that might lead to serious morbidities.^[1]^ It is often complicated by acute pyelonephritis that is characterized by an acute onset, serious illness, and nonspecific clinical manifestations. It can easily be misdiagnosed, and the prognosis might be delayed when treatment is delayed, leading to bacteremia and renal scarring, or even renal insufficiency that might require nephrectomy, causing a great harm to affected children.^[2,3]^ In this article, 2 cases of children with acute pyelonephritis complicated with renal abscesses were retrospectively collected and described. Both cases presented with nonspecific manifestations and a systemic inflammatory reaction. Moreover, we conducted a literature review to summarize the clinical characteristics, diagnosis, treatment, and prognosis of similar cases to improve clinical practice.

## 2. Case presentation

### 2.1. Case one

**Figure 1.**
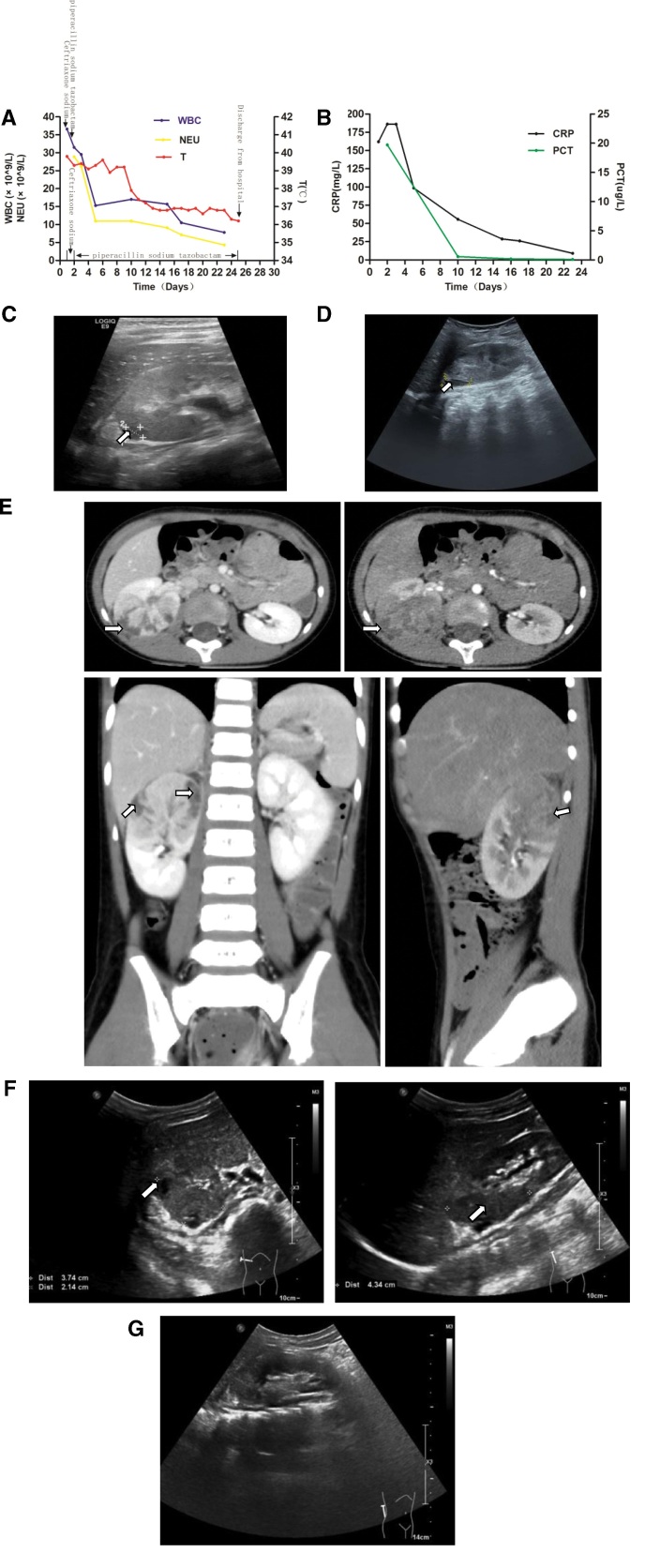
The changes of clinical indexes, kidney B-ultrasound, computed tomography (CT) before, and after treatment in case one. (A) Changes of white blood cell counts, neutrophil counts, and body temperature in case one. The white blood cell counts and neutrophil counts were significantly increased on admission, and the body temperature was repeatedly high, which decreased to normal after anti-infection treatment. (B) Changes of CRP and PCT in case one. The levels of CRP and PCT were significantly increased on admission, and went back to normal after anti-infection treatment. (C, D, F, and G) Renal ultrasonography was performed on the 2nd, 10th, 15th, and 23rd day after admission for case one. The abscesses of the right kidney were measured as 0.94 cm × 0.93 cm, 1.53 cm × 0.82 cm, 3.7 cm × 2.1 cm × 4.3 cm (arrow), and the abscesses were completely subsided on day 23. (E) Enhanced CT scan performed on day 6 of admission showed abnormal enhancement in the upper pole and middle of the right kidney, suggesting pyelonephritis with renal abscess formation (arrow). CRP = C-reactive protein, PCT = procalcitonin.

A 4-year-old girl presented at Xiamen Children’s Hospital with fever, vomiting, and abdominal pain on June 7, 2021. Fever occurred 1 day ago, with a peak of 39.8°C. Half a day ago, vomiting was associated with paroxysmal abdominal pain that was relieved yellow watery mucus-free stool. Other manifestations also included frequent micturition, urgency, pain, dysuria, hematuria, and foamy urine, with no recent history of trauma, contacting other patients or traveling abroad. Physical examination showed body temperature to be 38.9°C, with stable vital signs. Other manifestations also included painful expression, forced posture, slight tension of abdominal muscles, refusal to press, total abdominal tenderness, and no rebound pain, Laboratory evaluation showed leukocytosis and inflammatory markers were significantly increased (white blood cell count [WBC] 31.48 × 10^9^/L, neutrophil count [NEU] 28.88 × 10^9^/L, C-reactive protein [CRP] 186.18 mg/L, procalcitonin 19.71 μg/L) (Fig. 1A and B). Urine analysis showed that protein was 2+ mg/L, but no white or red blood cells were found. Biochemical parameters were also assessed (creatine kinase-MB 72 U/L, brain natriuretic peptide 265 pg/mL, creatinine 48 μmol/L, and urea nitrogen 4.5 mmol/L). Stool examination, erythrocyte sedimentation rate (ESR), and tumor markers were normal. Moreover, microbiological tests, include blood, urine, and fecal cultures. Since the child had no previous history of specific diseases, immune assessment assays were conducted. The results showed that the immunoglobulin level was low, with IgA 0.463 g/L (normal value 0.52–2.16 g/L), IgG 5.74 g/L (normal value 6.09–12.85 g/L), and IgM 0.606 g/L (normal value 0.67–2.46 g/L). On the other hand, T cell subsets were normal.

On the second day, urinary ultrasound detected the right renal abscess (0.94 cm × 0.93 cm in size, Fig. 1C). After admission, intravenous ceftriaxone sodium (80 mg/kg/d 6.8) was given, but the clinical manifestations and inflammatory indicators did not improve. Therefore, ceftriaxone was replaced by piperacillin sodium tazobactam (337.5 mg/kg/d 6.8–6.30). Complete abdominal computed tomography (CT) enhancement on day 6 after treatment showed right pyelonephritis with renal abscess formation (Fig. 1E). After 1 week of treatment, the body temperature dropped to be normal and stable. On the 10th, 15th, and 23rd days, renal ultrasound examination was performed to monitor the size of right renal abscess (Fig. 1D and F), which completely subsided (Fig. 1G) and the patient was discharged from hospital.

### 2.2. Case two

**Figure 2.**
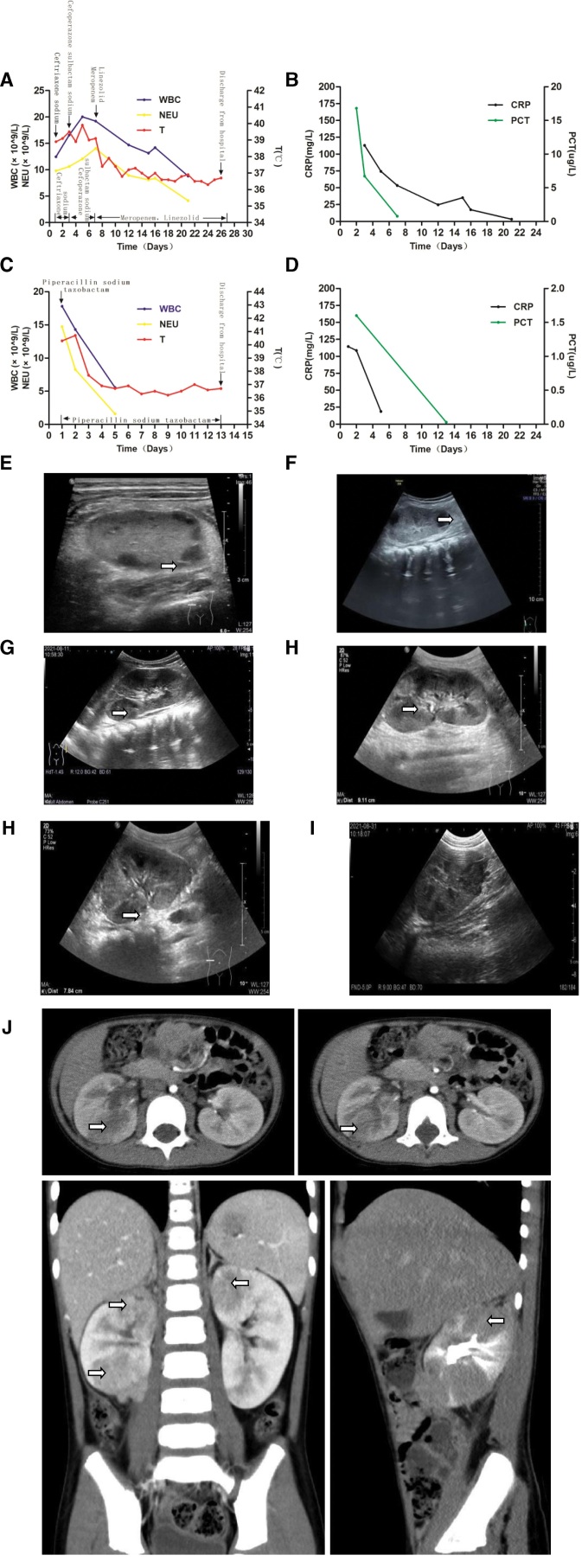
The changes of clinical indexes, kidney B-ultrasound, computed tomography (CT) before, and after treatment in case two. (A and C) Changes of white blood cell counts, neutrophil counts, and body temperature at the first (A) and second (C) hospitalization in case two. The white blood cell counts and neutrophil counts were significantly increased on admission, and the body temperature were high, which returned to normal after anti-infection treatment. (B and D) Changes of CRP and PCT at the first (B) and second (D) hospitalization in case two. The levels of CRP and PCT were significantly increased on admission, and decreased to normal after anti-infection treatment. (E–I) Renal ultrasonography was performed in case two on the 4th, 9th, 12th, 18th, and 24th day after the first admission. The abscesses of the right and left kidney were measured as 0.65 cm, 2.0 cm × 1.4 cm, 2.0 cm × 1.3 cm, 0.7 cm, and 0.8 cm (arrow). (J) Enhanced CT scan performed in case two on day 20 of the first admission showed bilateral pyelonephritis with right kidney protrusion (arrow). CRP = C-reactive protein, PCT = procalcitonin.

A 4-year-old boy presented to Xiamen Children’s Hospital with fever and abdominal pain for 2 days on July 30, 2021. Fever occurred 2 days ago, with a peak of 39.0°C. Paroxysmal abdominal pain, vomiting once, with no diarrhea, no frequent urination, urgency, pain, hematuria, and foam urine were also reported. Routine blood tests showed WBC 14.65 × 10^9^/L, NEU 11.85 × 10^9^/L, CRP 246.7 mg/L, and the colored Doppler ultrasound of gastrointestinal tract was normal. The patient had a history of urinary tract infection (UTI) at the age of 1 year and 6 months. Physical examination showed body temperature to be 39.0°C, with stable vital signs. He complained of abdominal distention with tenderness, and no rebound pain. In-hospital laboratory evaluation showed leukocytosis and elevated inflammatory markers (WBC 12.47 × 10^9^/L, NEU 9.80 × 10^9^/L, CRP 202.43 mg/L; procalcitonin 16.81 μg/L; ESR 74 mm/hr) (Fig. 2A and B). Urine routine showed a pathological cast + μL, occult blood 3 + mg/L, red blood cells 3–5/HP, white blood cells 5–10/HP, and protein 2+. The routine, biochemical, ESR, tumor markers, anti-O, humoral immunity, and T cell subsets were all normal. Moreover, microbiological tests, like blood, urine, and fecal cultures, G test, GM test, T-Spot, were all negative. Urinary color Doppler ultrasound indicated diffuse damage of both kidney and nephritis was suspected, with a slightly thickened bladder wall.

After admission, bacterial infection was considered due to significantly elevated inflammatory markers. Ceftriaxone (80 mg/kg/d 7.30–7.31) was given intravenously, but the symptoms did not improve. After reexamination, the inflammatory index was higher than before. Two days later, the plain CT scan of the whole abdomen showed that both kidneys were enlarged and uneven in density, suggesting inflammatory changes. Ceftriaxone was replaced by cefoperazone sulbactam sodium (240 mg/kg/d 8.1–8.5). On the 4th, 9th, 12th, 18th, and 24th day after treatment, continuous renal ultrasound examination showed that a decreasing pattern in the size of the renal abscess was 0.65 cm (Fig. 2E), 0.8 cm, 2.0 cm × 1.4 cm (Fig. 2F), 2.0 cm × 1.3 cm (Fig. 2G), 0.7 cm, 0.8 cm (Fig. 2H), until it disappeared, with only right renal parenchyma inflammation on the 24th day (Fig. 2I). On the 20th day, CT enhancement of the whole abdomen showed bilateral pyelonephritis, and the right kidney was prominent (Fig. 2J). One week after treatment, the inflammatory index improved, but the body temperature did not. Therefore, the macro gene detection in blood and urine was approached to clarify the etiology. Moreover, cefoperazone sulbactam sodium was replaced by meropenem (60 mg/kg/d 8.6–8.23) combined with linezolid (30 mg/kg/d 8.6–8.23). Combined with the pathogenic results of the macro gene detection in blood and urine (Table 1), amoxicillin clavulanate potassium (685.5 mg/kg/d 8.24–10.5) combined with nitrofurantoin (6.66 mg/kg/d, 8.24–10.5) were prescribed after discharge.

**Table 1:** mNGS report of bacteria and virus in blood and urine.

Pathogenic microorganism	Blood mNGS (sequence number)	Urine mNGS (sequence number)
Bacteria	*Escherichia coli* (976)	*Enterococcus faecalis* (45)
DNA virus	Epstein-Barr virus (7) Human cytomegalovirus (3)	JC Polyoma virus (107)
Fungus	Not detected	Not detected
Parasite	Not detected	Not detected
Mycobacterium	Not detected	Not detected
Drug resistance gene	Not detected	Not detected
Suspected background microorganism	Not detected	*Staphylococcus pasteurelli* (496) *Staphylococcus epidermidis* (172) *Fingoldelia macros* (19) *Brevibacterium casei* (5)

mNGS = metagenomic next-generation sequencing.

After treatment, antibiotics were stopped after the routine urine test and urinary color Doppler ultrasound were normal on October 5, 2021. However, 1 week later (October 12, 2021), fever and abdominal pain recurred in the child. Color ultrasound of urinary system suggested recurrence of renal abscess, and was hospitalized again. Laboratory evaluation showed leukocytosis and elevated inflammatory markers (Fig. 2C and D). He was given intravenous piperacillin sodium tazobactam (337.5 mg/kg/d) for 2 weeks and was discharged after improvement. Furthermore, considering that the child suffered from renal abscess twice in a short time, the excretory urography was performed with the consent of family members to evaluate whether there are urinary system abnormalities such as ureteral obstruction and vesicoureteral reflux. This indicated that the right vesicoureteral reflux was grade II. Renal static imaging (dimercaptosuccinic acid) showed pyelonephritis, right kidney scar, and impaired right renal function (71.68% for left kidney and 28.32% for right kidney). Urinary magnetic resonance imaging/MRU showed multiple wedge-shaped diffusion-restricted shadows in the parenchyma of both kidneys indicating inflammation. It also showed a larger left kidney (the upper and lower diameters of the left kidney were about 9.0 cm long and the upper and lower diameters of the right kidney are about 6.7 cm long).

### 2.3. Literature review

With the keywords of “pediatric” or “children” or “child” and “kidney abscess” and “fever” and “vomiting” and “abdominal pain,” the database of China How Net, Wan fang, VIP, and PubMed were searched. The search strategy was conducted in December 2022, 2 Chinese article and 32 worldwide articles were retrieved. Finally, a total of 192 cases with renal abscesses were selected from 20 articles (2 of which described their cases within the same center) after title and abstract screening.^[1,2,4–21]^ The results of literature review are summarized in Table 2. The most common clinical manifestation is fever (94.2%, n = 181/192), abdominal pain (41.7%, n = 80/192), vomiting (42.1%, n = 81/192), 80.1% (n = 117/146) and 83.1% (n = 108/130) of the patients had a common increase in WBC and CRP. The most common isolated microorganism was *Escherichia coli* infection in 77 cases (74%, n = 77/104). *Staphylococcus aureus* is a less common pathogenic microorganism, only 6.7% (n = 7/104). Other microorganisms include *Klebsiella pneumoniae, Pseudomonas aeruginosa, Enterococcus, Proteus mirabilis, Salmonella, Streptococcus vermicelli*, and *Enterobacter aerogenes*. The blood culture positive rate was 6% (n = 10/167), including *E coli* 40% (n = 4/10) and *S aureus* 20% (n = 2/10). The positive rate of pus culture was 90.5% (n = 38/42). The common isolated microorganism was *E coli* (12/28, 10 cases of unknown pathogens were excluded) and *S aureus* (15/28). One case with *Proteus mirabilis*; 73.4% (n = 141/192) of children received only antibiotic treatment and 22.9% (n = 44/192) of cases treated by percutaneous drainage. Only 3% (n = 6/192) of children received surgical treatment; 0.5% (n = 1/192) of children received anti-tuberculosis treatment; 40% (n = 71/192) of cases combined with abnormal urinary tract development and upper respiratory infection. Forty-nine cases used micturating cystourethrogram for follow-up imaging, and 69.4% (n = 34/49) of children were found to have reflux. One-third (n = 6/18) of children had reduced kidney function (Table 2).

**Table 2:** Summary of literature review findings.

Literature	Cases	Fever (ratio (%))	Vomiting (ratio (%))	Abdominal pain (ratio (%))	Other clinical manifestations	Underlying etiology	Inflammatory indicators: leukocyte, CRP, PCT	Urine routine	Urine culture	Blood culture	Pus culture	Renal ultrasound	Computed tomography	Renal abscess location	Combined with urinary malformation	Treatment	Follow-up: renal scar and renal function
Luo TH et al^[4]^	5	4	1	3	Low back pain 1 scoliosis 1 headache 1	UTI 3, unknown etiology 2	Leukocyte, CRP, PCT increased 4, normal 1	Leukocyte positive 2, urine protein positive 1 Normal 2	*E coli* 1, negative 4	Negative 5	–	5	5	Left 3, Right 2	No	Antibiotics 5	Renal scar and decreased renal function 1, normal 4
Xu YQ et al^[5]^	1	1	1	1	Diarrhea	–	Leukocyte, CRP increased, platelet decreased	Urine leukocytosis and red blood cells were elevated	Negative	Negative	–	1	1	Left	–	Antibiotics	CT showed reduced left kidney
Chen CY et al^[6]^ Chen CY et al^[7]^	17	17	8	4	Diarrhea 1 abdominal pain 12	–	Leukocyte, CRP increased 17	Urine leukocytosis 10, red blood cells elevated 6	*E coli* 7, *Klebsiella pneumoniae* 1, negative 9	Negative 17	Total 4, *E coli* 2, *Staphylococcus aureus* 1, negative 1	17	17	Left 3 Right 12 Bilateral 2	Right side III–V Vesicoureteral reflux 3 (VUR)	Antibiotics 13, antibiotics + ultrasound-guided percutaneous abscess drainage 4	–
Cheng CH et al^[2]^	45	45	20	16	Hypochondriac pain 14 peritoneal signs 4 diarrhea 3 claudication gait 1 shock and pleural effusion 1	Acute lymphoblastic leukemia 1, normal 44	–	–	Total 45, *E coli* 24, *Klebsiella pneumoniae* 1, *Pseudomonas aeruginosa* 1, *Staphylococcus aureus* 1, negative 18	Total 45, *E coli* 1, negative 44	Total 3, *E coli* 2, *Staphylococcus aureus* 1	–	45	–	Vesicoureteral reflux 18 (total 42) Grades one to three 13	Antibiotics 42, antibiotics, CT-guided drainage 3	Renal scar 21 (total 23)
Zhang X et al^[1]^	17	14	5	1	Loss of appetite 6 changes in urination 5 bladder irritation 4 oliguria 2 edema 1 abdominal mass 1 abdominal tenderness or spasm 1	–	Leukocyte increased 12, CRP increased 15, PCT increased 1	Leukocytosis 11 Erythropoietic 5 Protein-positive 5	Total 17, positive 7, negative 10	Total 17, positive 0, negative 17	Total 10, positive 9, negative 1	17	8 (MRI 14, MCU 4)	–	Abnormal genitourinary system 10 abnormal ureter morphology (stenosis, dilation, or distortion) 7 repeated UTI 4 nonfunctional kidney or dysplasia 3 vesicoureteral reflux 2 kidney stones 2 renal cysts 1	Antibiotics 6, Antibiotics, Surgery 10 (including nephrectomy 2) Anti-tuberculosis 1	–
Bakker AM et al^[8]^	2	2	2	2	Hypertension	–	Leukocyte, CRP increased 2	Normal 2	*Enterococcus* 1, negative 1	Negative 2	–	2 (negative)	0 (MRI 2)	Left 1 Right 1	No	Antibiotics 2	Decreased renal function 1 Normal 1
Seguias L et al^[9]^	36	34	24	13	Hematuria 21 dysuria 9 touching mass 1	–	Leukocyte increased 25 (total 35) CRP increased 21 (total 25)	Leukocyte or nitrite positive 23	Positive 19 (*E coli* 11, *Staphylococcus aureus* 4, Methicillin-sensitive *Staphylococcus aureus* 2, *Salmonella, Enterococcus, Streptococcus viridis, Enterobacter aerogenes, Proteus mirabilis*)	Positive 6 (*E coli* 3)	Total 8, positive 7 (*E coli* 4, methicillin-sensitive *Staphylococcus aureus* 1, methicillin-resistant *Staphylococcus aureus* 1)	29	35	–	Total 36 Abnormal genitourinary system 11 Vesicoureteral reflux 3, Structural bladder abnormalities 3, Repeated set system 2, Neurogenic bladder 2, Posterior ureteral valve 1, Pyeloureteral junction obstruction 1, Urethral stricture 1	Antibiotics 24, Antibiotics, percutaneous drainage 12	–
Linder BJ et al^[10]^	16	14	9	8	Flank pain 9 (56%) dysuria 5 (31%) hematuria 1 (6%)	UTI 13 Hematogenous seed 1 Unknown 2	Leukocyte increased 13 CRP increased 8	–	*E coli 10 (total 13*)	–	*Staphylococcus aureus* 1	–	–	–	Class I VUR 2 (total 10)	Antibiotics 12, Antibiotics; percutaneous drainage 4	–
Buschel H et al^[11]^	14	13	6	12	Dysuria 14% (n = 2) hematuria 14% (n = 2)	UTI 6 Hematogenous spread 7 Unknown 7	Leukocyte increased 10 CRP increased 12	–	*E coli* 4, MRSA 1, *Pseudomonas aeruginosa* 1	MRSA 1, Penicillin resistant *Staphylococcus aureus* 1	*E coli* 2, MRSA 4, Penicillin resistant *Staphylococcus aureus* 2	12	11	Left 4 Right 8 Bilateral 2	Pyelonephritis in infancy 1	Antibiotics 7; Antibiotics; percutaneous drainage 5, Antibiotics; open drainage 2	Renal scar 1 (total 3)
Martonosi ÁR et al^[12]^	1	1	–	–	Bilateral nonsuppurative conjunctivitis, weight loss, muscle pain, general discomfort, cough	No	Leukocyte, CRP increased	Normal	Negative	Negative	*E coli*	1	0 (MRI 1)	Right	Left renal upper pole 20 × 16 mm and 8 mm cyst	Antibiotics; open surgical drainage	–
Constantine S et al^[13]^	1	1	–	1	Lethargy, weight loss, cough	–	Leukocyte normal, CRP, PCT not mentioned	–	–	–	*Staphylococcus aureus*	–	1	Right 1	Enuresis, UTI, chest infections, skin abscesses	Antibiotics, percutaneous drainage, nephrectomy	–
Huq S et al^[14]^	1	1	1	1	Nausea	–	Leukocyte, CRP increased	Leukocytes and erythrocytes were elevated, nitrite and urine protein were positive	Negative	–	–	1	1	Right	Hernia operation	Antibiotics	–
Taksande AM et al^[15]^	1	1		1	Cough, anemia	–	Leukocyte increased	Normal	Negative	Negative	–	1	1	Left	Sickle cell anemia “SS” type	Antibiotics	–
Cancelinha C et al^[16]^	2	1	1	2	Myalgia 1 chills 1 runny nose 1 cough 1 pale 1, left abdominal mass 1	–	Leukocyte increased 2, CRP, PCT increased 1	Urinary leukocyte increased 1	Negative 2	Negative 2	Case 2 positive (*Proteus mirabilis*)	2	2	Left 2	Case 2 Mild beta thalassemia	Antibiotics 1, Antibiotics, surgery 1	Renal scarring 2, Normal renal function 2
Baradkar VP et al^[17]^	1	1	No	1	–	–	Leukocyte increased	Protein positive, pus cells increased	Negative	–	*E coli*	1	No	Left	renal calculus	Antibiotics, percutaneous drainage	–
Fernandes RC et al^[18]^	3	3	No	3	Left abdominal mass 1 weakness 1	–	Leukocyte increased 2	Normal 3	Negative 2, no culture 1	Negative 2, no culture 1	*Staphylococcus aureus* 2, negative 1	3	2	Left 2, Right 1	Pyeloureter dilatation 1	Antibiotics, percutaneous drainage 2; Antibiotics, open drainage 1	–
Pampinella D et al^[19]^	1	1	No	1	Testicular radiation pain	No	Leukocyte, CRP increased	Leukocytes and erythrocytes were elevated; protein-positive	–	–	Methicillin-resistant *Staphylococcus aureus*	1	1	Right	–	Antibiotics, nephrectomy	–
Jiménez M et al^[20]^	20	20	9	4	Lower urinary tract symptoms 5 gross hematuria 1 diarrhea 5	–	Leukocyte, CRP increased 16	18	18, *E coli* 14, *Pseudomonas* 1, *Klebsiella* 2, *Staphylococcus aureus* 1	–	–	15, unilateral 14, bilateral 1	7, unilateral 6, bilateral 1	–	UTI 1, Dysurination 6	Antibiotics 19, Antibiotics, percutaneous drainage 1	Decreased renal function 4 (total 9)
Wang YT et al^[21]^	8	8	3	4	Poor activity 1, poor appetite 3, URI 1	–	Leukocyte, CRP increased 8	Urinary leukocyte increased 8	*E coli* 6	1	–	8	8	Left 3 Right 4 Bilateral 1	Vesicoureteral reflux 3	Antibiotics 7, Antibiotics; percutaneous drainage 1	Renal scar 5 (total 7)
Summary	192	181 (94.2%)	81 (42.1%)	80 (41.7%)	–	–	Leukocyte increased 117 (total 146, not mentioned 46), CRP increased 108 (total 130, not mentioned 62), PCT increased 6 (total 24, not mentioned 168)	Urine leukocyte increased 77; urine red blood cell increased 32, urine protein positive 27	Positive 104	Positive 10	Positive 38	Total 116, not mentioned 16	Total 147, not mentioned 16	Left 21 Right 32 Bilateral 5 Not mentioned 134	Developmental abnormalities of urinary tract and UTI 71	Antibiotics 141, Antibiotics; percutaneous drainage 44, Antibiotics; surgery 6, Anti-tuberculosis 1	Renal scar 34 (total 49) Not mentioned 143 Decreased renal function 6 (total 18) Not mentioned 174

Note: ^[6,7]^ The kidney abscess data described in these 2 articles came from the same center.

“–” = not mentioned in the literature, CRP = C-reactive protein, MCU = micturating cysto urethrogram, MRI = magnetic resonance imaging, MRSA = methicillin resistant *Staphylococcus aureus*, PCT = procalcitonin, URI = upper respiratory infection, UTI = urinary tract infection.

## 3. Discussion

The exact etiology and pathogenesis of renal abscess in children are not clear. Estimates show that children with renal abscess often have abnormal ureters (41.2%), renal dysplasia or nonfunctional kidneys (17.6%), vesicoureteral reflux (11.8%), and other genitourinary system abnormalities.^[1,10]^ Moreover, 23.5% of these children usually have a history of repeated lower UTIs,^[22]^ suggesting that these events might predispose to developing renal abscesses. We presented case two with a history of UTIs, and 2 renal abscesses. Dimercaptosuccinic acid showed pyelonephritis, right kidney scar, and impaired function. Considering the possibility of urinary tract abnormalities such as abnormal ureter and vesicoureteral reflux, the excretory urography was improved, and the results showed that the right vesicoureteral reflux was grade II, which indicated our expectations that were based on physical and clinical evaluation. However, case one had no previous history of UTIs and other systemic infections. Because the family members refused to take cystourethrography examination, it was not clear whether there were urogenital malformations, like vesicoureteral reflux. However, its humoral immune indexes (IgA, IgG, IgM) were low, suggesting that immune assays might be used to predict renal abscesses in these children. Therefore, assessment of immune assays should be considered in children with suspected renal abscesses.

Renal abscess might develop secondary to different routes, including ascending urinary tract and hematogenous infections, and the spread of inflammation from the adjacent organs. Different routes of infection might introduce various pathogenic bacteria. Upward infection is often associated with urogenital malformation or repeated UTIs. The main pathogenic bacteria are gram-negative bacteria, mainly *E coli*. Moreover, Staphylococcal infection is the most common cause of hematogenous spread.^[23,24]^ Abscess is often complicated by a severe inflammatory reaction, but the sensitivity rate of routine blood culture is extremely low. Our literature review findings demonstrated that in children with renal abscess, the blood culture positive rate was only 6%, suggesting that hematogenous dissemination may not be the main mechanism of childhood renal abscess, as indicated in our 2 cases. After 1 week of treatment with third generation cephalosporin, no improvement was noticed in case two. Therefore, metagenomic next-generation sequencing (mNGS) for detecting blood and urine pathogens was carried out. Even after 1 week of treatment with third generation cephalosporins, *E coli* and *Enterococcus faecalis* were still detectable, which is consistent with previous results that *E coli* is the main pathogen of renal abscess caused by upward infection, providing a pathogenic reference for subsequent antibiotic sequential treatment. Moreover, using mNGS technology might be helpful to clarify the etiology and enhance choosing the most sensitive treatment regimen.

mNGS is a new tool that can directly perform high-throughput sequencing on nucleic acids in samples to identify potential pathogens quickly and accurately.^[25]^ Various types of specimens can be tested, including cerebrospinal fluid, pleural, peritoneal and joint cavity effusions, alveolar washings, sputum, concentrated juice, blood, and tissues. The modality is less affected by antibiotic use,^[26]^ which improves is sensitivity, and is recommended for the diagnosis of acute and critical conditions and detecting difficult infections.^[27]^ Recently, many studies have found that mNGS plays an important role in pathogen detection at multiple infected sites, covering central nervous system infections, liver tuberculosis, joint cavity effusion infection, adrenal abscess, and special pathogen pneumonia.^[28–34]^ However, there is a lack of data regarding the efficacy of mNGS detection in identifying the causative organism of renal abscess. As far as we know, this is the first report to do so.

The clinical manifestations of renal abscess in children lack specificity. Fever is the most common manifestation, and some children can show typical urinary symptoms such as foam urine, hematuria, turbid urine, frequent urination, urgency, and pain. However, for older children, there are few typical urinary symptoms, but atypical symptoms such as lumbago, abdominal pain, nausea, and vomiting might also be detected.^[4,7,9]^ Moreover, physical examination might show abdominal tenderness, rebound pain, or the presence of an abdominal mass. Therefore, the diagnosis of this condition might be misleading and further measures are required to establish a proper differential diagnosis.

The 2 children admitted to our hospital were mainly characterized by fever, vomiting, and abdominal pain. They were diagnosed as having underlying digestive diseases early, and excluding surgical acute abdomen was established accordingly. A final diagnosis acute pyelonephritis complicated with renal abscess was then established after additional measures were conducted. Children with atypical clinical symptoms are easily misdiagnosed. Without the assistance of B-ultrasound, CT, and other imaging modalities, it is difficult to make a clear diagnosis by clinical manifestations and physical examination only. CT examination is more advantageous in the diagnosis of renal abscess,^[16]^ but in this clinical case, B-ultrasound and CT have the same efficacy in evaluating renal abscesses. Moreover, since B-ultrasound is simple and has no radiation, it can be used as a clinical follow-up evaluation method for the treatment course planned for renal abscesses.

Gram-negative bacilli are the main pathogenic bacteria of acute pyelonephritis and renal abscess in children, among which *E coli* is the most common.^[4,7,18,19]^ Therefore, second- or third-generation cephalosporins are often used as the first choice antibiotics for managing renal abscesses. However, our cases did not respond well to these types of antibiotics. Two cases were treated with ceftriaxone sodium for anti-infection in the early stage, but the therapeutic effect was poor. Case one was replaced with piperacillin sodium tazobactam sodium for anti-infection, and the renal abscess completely disappeared after 3 weeks of treatment. In case two, body temperature was still high 1 week after replacement of cefoperazone sulbactam sodium treatment. Finally, the clinical symptoms returned normal only after meropenem combined with linezolid were administered. Considering the harmfulness and destructiveness of renal abscess in children, antibiotic descending step therapy is the first choice in clinic. Initial extended spectrum antibiotics were given to quickly control clinical symptoms, and then the antibiotic grade was lowered according to the therapeutic effect and drug sensitivity test. Clinical analysis showed that the etiology of children with complicated upper UTI was mainly gram-negative bacilli, and the proportion of extended-spectrum β-lactamases-positive strains was high.^[35]^ The resistance rate of penicillin, the first and second generation cephalosporins is high, and piperacillin tazobactam has high sensitivity to bacteria and good anti-infection effect. The clinical treatment results of 2 cases in this paper were consistent with the above clinical analysis.

Urine mNGS suggested *E faecalis* infection (Table 1), indicating the poor efficacy of antibiotic therapy. Accordingly, targeting gram-positive bacteria infection and strengthen the anti-gram-positive bacteria treatment should be considered. In addition, clinicians should be alert about tuberculosis and fungal infections, and pay attention to improving purified protein derivative, T-spot, acid-fast bacilli culture, G test, and GM test.^[1,36]^ At present, the course of antibiotic treatment for renal abscess remains to be determined. However, it has been suggested that antibiotic treatment should be administered for 4 to 6 weeks even if a surgical intervention was approached.^[37,38]^ In this study, the course of antibiotic treatment in case one and two was 3 and 9 weeks, respectively.

The course of antibiotic treatment should be determined according to the follow-up results. Most renal abscesses can completely resolve after the administration of a full course of specific antibiotics with conservative treatment, as shown in our cases. However, for children with large renal abscesses or poor conservative treatment, surgical treatment might be needed. It can be done mainly through B-ultrasound-guided percutaneous abscess drainage and nonfunctional nephrectomy. At present, there is no standard for the surgical removal of a renal abscess. Managing renal abscesses was divided into groups based on their diameters. It was suggested that renal abscesses larger than 3 cm can be treated by percutaneous abscess puncture, and those smaller than 3 cm can be treated by conservative and antibiotic therapy.^[4,19]^ Some considered the risk of surgical treatment and chose 4 cm as the standard.^[1]^ Therefore, the specific standard for managing renal abscesses should be determined clinically.

## 4. Conclusions

The clinical symptoms of renal abscess complicating acute pyelonephritis in children are not typical, which is easy to cause missed diagnosis and misdiagnosis. Clinicians should suspect the presence of a renal abscess even in the absence of clinical and laboratory indicators. Improving imaging examinations such as B-ultrasound and CT enhancement can help confirm the diagnosis. Detecting mNGS in blood and urine can also be considered in cases of negative blood and urine cultures, to help find the etiology and guide clinical medication. If the abscess is small in size (diameter < 3 cm), timely treatment with specific broad-spectrum antibiotics can improve the outcomes. If the response is poor or the abscess size is large, surgical treatment such as percutaneous abscess puncture and drainage or nephrectomy can be performed when necessary.

## Author contributions

**Conceptualization:** Qian Shen, Xiaoliang Lin.

**Data curation:** Zhuqin Zhan.

**Formal analysis:** Zhuqin Zhan.

**Funding acquisition:** Zhuqin Zhan.

**Investigation:** Jinhua Zeng, Jianying Liao.

**Methodology:** Dequan Su.

**Project administration:** Zhuqin Zhan.

**Validation:** Guangbo Li.

**Writing – original draft:** Zhuqin Zhan.

**Writing – review & editing:** Qian Shen, Xiaoliang Lin.
